# Multi-Agent DRL for Air-to-Ground Communication Planning in UAV-Enabled IoT Networks

**DOI:** 10.3390/s24206535

**Published:** 2024-10-10

**Authors:** Khalid Ibrahim Qureshi, Bingxian Lu, Cheng Lu, Muhammad Ali Lodhi, Lei Wang

**Affiliations:** Key Laboratory for Ubiquitous Network and Service Software of Liaoning Province, School of Software, Dalian University of Technology, Dalian 116024, China; khalidibrahim84@gmail.com (K.I.Q.); lc196@mail.dlut.edu.cn (C.L.); alilodhi30@gmail.com (M.A.L.)

**Keywords:** IoUAVs, MADRL, SDN

## Abstract

In this paper, we present a novel method to enhance the sum-rate effectiveness in full-duplex unmanned aerial vehicle (UAV)-assisted communication networks. Existing approaches often couple uplink and downlink associations, resulting in suboptimal performance, particularly in dynamic environments where user demands and network conditions are unpredictable. To overcome these limitations, we propose a decoupling of uplink and downlink associations for ground-based users (GBUs), significantly improving network efficiency. We formulate a comprehensive optimization problem that integrates UAV trajectory design and user association, aiming to maximize the overall sum-rate efficiency of the network. Due to the problem’s non-convexity, we reformulate it as a Partially Observable Markov Decision Process (POMDP), enabling UAVs to make real-time decisions based on local observations without requiring complete global information. Our framework employs multi-agent deep reinforcement learning (MADRL), specifically the Multi-Agent Deep Deterministic Policy Gradient (MADDPG) algorithm, which balances centralized training with distributed execution. This allows UAVs to efficiently learn optimal user associations and trajectory controls while dynamically adapting to local conditions. The proposed solution is particularly suited for critical applications such as disaster response and search and rescue missions, highlighting the practical significance of utilizing UAVs for rapid network deployment in emergencies. By addressing the limitations of existing centralized and distributed solutions, our hybrid model combines the benefits of centralized training with the adaptability of distributed inference, ensuring optimal UAV operations in real-time scenarios.

## 1. Introduction

To address the growing data traffic demand, service providers are increasingly adopting network densification strategies. This approach involves the deployment of numerous dense base stations (BSs) and smaller heterogeneous cell BSs, which are critical for the evolution of future networks. The primary objective of enhancing BS diversity and density is to improve network capacity and extend coverage, thereby accommodating the escalating data traffic requirements [1,2]. However, ultra-dense networks present challenges such as high costs, limited scalability, and extended deployment times [3]. As a result, they may not be ideal for applications requiring immediate response, such as search and rescue operations during natural disasters [4].

In contrast, unmanned aerial vehicles (UAVs) offer a more effective solution for this issue. Unlike fixed base stations, which are stationary and limited in height, UAVs are mobile and can operate at higher altitudes, enhancing the potential for line-of-sight links [5]. When deployed strategically, UAVs are also cost-effective for providing extensive services [6]. Furthermore, UAV-based networks can implement in-band full-duplex communication, allowing for simultaneous transmission and reception over the same frequency [7,8]. This capability can significantly improve network spectral efficiency, leading to increased interest in UAV-based communication research [9].

Traditional wireless communication approaches, which rely on fixed infrastructure, differ significantly from UAV-based communication. In UAV-based systems, the communication link between UAVs and ground-based users (GBUs) is not constant due to the mobility of UAVs, resulting in a highly dynamic network topology and increased uncertainty in link stability. Consequently, the processes of user association, transmission power selection, channel selection, and network coverage require different considerations. Many studies [10,11] assume that in UAV-GBU communication, a GBU remains associated with the same UAV for both uplink and downlink communication. This model is appropriate when the same power levels are used for uplink and downlink. However, in networks where uplink and downlink power levels differ, this approach is not effective.

**Figure 1:**
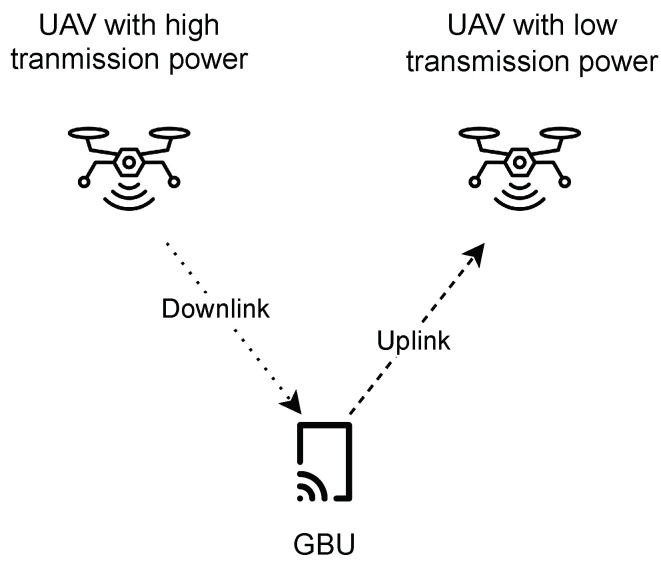
Separate uplink and downlink association in full-duplex communication for ground user.

For example, as illustrated in Figure 1, consider a GBU that wants to maximize its downlink performance by connecting to UAV 1, which provides a high downlink rate. However, for uplink communication, the GBU prefers to use a lower power level, so it connects to UAV 2, which is optimized for lower-power uplink transmissions. This scenario illustrates the challenge of managing separate UAV connections for uplink and downlink communications based on varying power levels and performance requirements, motivating the adoption of a decoupled association approach that optimizes overall communication performance.

Moreover, since UAVs operate on batteries, their ability to provide network services is limited in duration [12,13]. To reduce this limitation, adopting optimal strategies is essential. The association between UAVs and GBUs depends on transmission distance, necessitating that UAV trajectories align with association requirements. Therefore, optimizing both UAV-GBU association and UAV trajectory can enhance network performance. Several studies have explored UAV-assisted wireless networks to optimize power management and trajectory design for efficient data collection and transmission, such as UAV-based wireless-powered communication networks (WPCNs) that minimize the long-term average Age of Information (AoI) through clustering and power allocation strategies [14].

Furthermore, Software-Defined Networking (SDN) offers a robust framework for managing full-duplex communication due to its centralized control and dynamic resource management capabilities. SDN excels in mitigating both self-interference and interference from other users by providing real-time adjustments to network parameters such as transmission power and frequency allocation [15]. Its inherent flexibility and programmability allow for precise control over network resources, enhancing overall communication performance and efficiently addressing interference issues. This makes SDN an effective approach for optimizing network operations in complex communication environments.

While SDN provides a robust foundation for managing network resources and interference, optimizing complex scenarios such as multi-UAV networks requires advanced techniques. Deep Reinforcement Learning (DRL) frameworks are gaining traction for optimizing multi-UAV network problems [16]. DRL combines reinforcement learning (RL) with deep learning to handle complex environments where traditional RL methods may struggle [17]. In DRL, an agent interacts with the environment and makes decisions based on feedback in the form of rewards, aiming to maximize cumulative rewards through learning from its experiences. While DRL is effective for single-agent scenarios, our problem involves numerous possible trajectories, making it computationally intensive. Managing this complexity with a single-agent approach can be challenging. To address this, multi-agent DRL techniques extend the framework to multiple interacting agents, offering potential solutions for dynamic and complex environments [18,19]. This paper explores how DRL can be adapted to optimize multi-UAV network performance and addresses the unique challenges associated with applying DRL in this context.

The novelty and contributions of our work are as follows:

- To improve the system’s sum rate, we propose a novel approach that decouples uplink and downlink associations for GBUs within a full-duplex communication framework. Our contribution lies in formulating a comprehensive optimization problem that jointly optimizes both uplink and downlink associations alongside UAV trajectory design, aimed at maximizing the overall sum-rate efficiency of the network.
- To address the non-convexity of the formulated problem, we reformulated it as a Partially Observable Markov Decision Process (POMDP). In this approach, each UAV operates as an autonomous agent, making real-time decisions regarding user association and trajectory optimization based on its local observations, without requiring complete global information about the environment.
- To address the POMDP, we present a framework based on multi-agent deep reinforcement learning (DRL) that leverages centralized training and distributed execution to achieve near-optimal policy design. Through the application of the Multi-Agent Deep Deterministic Policy Gradient (MADDPG) [20] algorithm, this technique enables UAVs to learn optimal user association and trajectory control efficiently while operating in a decentralized manner, adapting dynamically to local conditions.
- The proposed solution is tailored for critical applications such as disaster response and search and rescue missions. Our work highlights the practical significance of using UAVs for rapid network deployment in emergency situations, emphasizing the real-world impact of our research.
- We address the limitations of existing centralized and distributed solutions by offering a balanced approach. Our hybrid model ensures optimal UAV operations by combining the benefits of centralized training with the adaptability of distributed inference.

The remainder of the paper is structured as follows: Section 2 reviews recent and relevant research in the field. Section 3 introduces the fundamental network model and outlines the problem formulation. In Section 4, we explain the principles of Multi-Agent Reinforcement Learning (MARL), convert our problem into a Partially Observable Markov Decision Process (POMDP), and present our proposed Multi-Agent Deep Reinforcement Learning (MADRL) solution. Section 5 describes the experimental setup and discusses the results. Finally, Section 6 provides the conclusions of the study.

## 2. Related Work

Various studies have proposed strategies for optimizing UAV deployment and communication in wireless networks.

The work by [21] explores a comprehensive framework for full-duplex decoupled user association in multi-tier cellular networks. By formulating a joint uplink and downlink user association problem, this study effectively demonstrates the potential of decoupled user association to enhance overall network performance despite the challenges posed by interference in full-duplex communications. Additionally, the authors in [22] present compelling arguments for decoupling downlink and uplink transmissions in cellular networks, highlighting its potential to significantly enhance network throughput, reduce outages, and lower power consumption at a lower cost.

The downlink-uplink decoupled user association in heterogeneous networks (HetNets) significantly enhances system capacity and frequency efficiency. This work presents an optimization scheme that incorporates a multiple region frequency allocation (MRFA) strategy to alleviate interference among users, paired with a convergent power control (CPC) algorithm to optimize transmit power, demonstrating improved system throughput and user performance [23]. Furthermore, the study on load balancing in heterogeneous cellular networks (HCNs) emphasizes the significance of decoupled downlink-uplink associations, revealing that employing differing association strategies for uplink and downlink can markedly improve joint uplink-downlink rate coverage, particularly under conditions of load imbalance [24,25].

Various studies have explored UAV trajectory optimization. The authors in [26] present a novel control algorithm utilizing Deep Deterministic Policy Gradient (DDPG) for two-dimensional trajectory design and power allocation in UAV wireless networks, optimizing energy efficiency and communication service rates. Additionally, the development of a 3D multi-UAV deployment framework integrates user association scheduling and power control using a block coordinate descent-based iterative algorithm [27].

Recent advancements have also focused on multi-agent deep reinforcement learning (MADRL) approaches for optimizing UAV trajectories. For instance, reference [28] introduces a multi-agent DRL-based scheduling algorithm to optimize charging time, phase shifts, UAV associations, and trajectories in multi-UAV, and multi-IRS networks. Another study proposes an inverse soft-Q learning algorithm for multi-IRS multi-user association in UAV communications, significantly improving energy efficiency and convergence [29].

The authors in [11] formulate the UAV localization and association problem as a submodular maximization problem under a matroid constraint, enabling a greedy approach with a performance guarantee of $1-\frac{1}{e}$. They also propose a heuristic greedy algorithm that achieves results in a few iterations, although it may not be optimal in all scenarios. Q-learning-based methods have also been explored in UAV communication networks. For instance, a reinforcement learning framework was proposed to optimize the 3D location and power of UAVs as aerial base stations (ABSs), prioritizing emergency users through a carefully designed reward function [30]. Their results demonstrated significant improvements in user coverage, aligning with the potential benefits of using Q-learning-based approaches such as MADQN in optimizing UAV performance.

While the aforementioned studies primarily address UAV trajectory and resource management, the investigation of full-duplex (FD) communication challenges has been a vital aspect in maximizing system capacity. Joint trajectory design, transmission scheduling, and power adjustment solutions have been proposed to address severe uplink interference caused by downlink transmissions [31,32]. The exploration of FD UAVs in uplink-downlink NOMA scenarios, focusing on simultaneous communication between sources and destinations via FD-aided UAVs over Nakagami-m channels, also contributes to the understanding of performance enhancements in UAV networks [33].

Lastly, a comprehensive survey of UAV-based communication networks covers critical aspects such as technologies, path planning, and power management strategies [34].

## 3. Network Model and Problem Formulation

### 3.1. Network Configuration and Deployment

**Figure 2:**
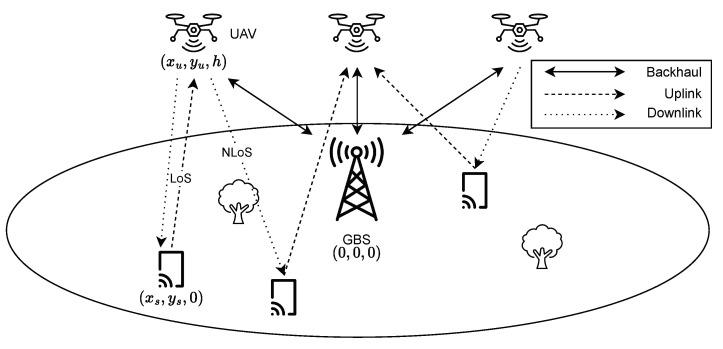
Network model illustrating the communication setup with one GBS, multiple UAVs serving as FBS, and GBUs. The figure depicts both LoS and NLoS links, as well as uplink and downlink transmissions between GBUs and UAVs.

We consider a software-defined Internet of UAVs (SD-IoUAVs) network, served by one fixed ground base station (GBS) and several UAVs functioning as flying base stations (FBSs), with both Line-of-Sight (LoS) and Non-Line-of-Sight (NLoS) links, as shown in Figure 2.

The set of FBSs, denoted as F, is given by F={1,2,3,…,F}, and the set of GBUs, denoted as U, is given by U={1,2,3,…,U}, where *F* and *U* represent the total number of UAVs and users, respectively. The GBS *G* is located at the origin, with coordinates ${\left(x,y,h\right)}_{G}=\left(0,0,0\right)$, where h is the altitude. For simplicity, the altitude of the GBS is considered negligible compared to that of the FBSs and is approximated as 0. In this network scenario, the locations of the GBUs are randomly distributed. The location coordinates of GBU *u* are given by $l_{u}=\left(x_{u},y_{u},0\right)$, and the location coordinates of FBS *f* are given by $l_{f}=\left(x_{f},y_{f},h\right)$. For our proposed solution, we assume a fixed altitude h for all UAVs. The geometric distance $d_{fu}$ between GBU *u* and FBS *f* can be calculated as:

$$d_{fu}=\sqrt{{\left(x_{f}-x_{u}\right)}^{2}+{\left(y_{f}-y_{u}\right)}^{2}+h^{2}}.$$

The UAVs can move with a maximum velocity of $\nu^{max}$ in the direction $\Phi_{f}=\left[0,2\pi \right]$.

### 3.2. Channel Model

In IoUAV networks, the air-to-ground (A2G) communication links between FBSs and GBUs can be categorized into LoS or NLoS depending on the environmental factors. The probability of a LoS link can be calculated as:

(1) $$P^{LoS}\left(t\right)={\left[1+b_{1}\exp\left(-b_{2}\left(\omega_{fu}\left(t\right)-b_{1}\right)\right)\right]}^{-1},$$

where $b_{1}$ and $b_{2}$ are constants related to the network environment (e.g., urban, rural), and $\omega_{fu}\left(t\right)$ is the elevation angle, which can be calculated as:

$$\omega_{fu}\left(t\right)=\frac{180}{\pi }\arcsin\left(\frac{h}{d_{fu}}\right).$$

With the LoS probability calculated, the probability of NLoS can be obtained as:

(2) $$P^{NLoS}\left(t\right)=1-P^{LoS}\left(t\right).$$

For LoS and NLoS links, the path loss between FBS *f* and GBU *u* can be calculated as:

(3) $$L_{fu}^{LoS}\left(t\right)=w_{fu}\left(t\right){ℵ}^{LoS},$$

(4) $$L_{fu}^{NLoS}\left(t\right)=w_{fu}\left(t\right){ℵ}^{NLoS},$$

where ${ℵ}^{LoS}$ and ${ℵ}^{NLoS}$ are attenuation coefficients for LoS and NLoS communication links, respectively. The power gain, $w_{fu}\left(t\right)$, is given by

$$w_{fu}\left(t\right)={\left(\frac{4\pi \mu }{c}d_{fu}\left(t\right)\right)}^{2},$$

where μ is the carrier frequency and *c* is the speed of light in free space. Using Equations (1)–(4), the average path-loss between FBS *f* and GBU *u* at time slot *t* can be obtained as:

(5) $$L_{fu}\left(t\right)=P^{LoS}\left(t\right)L_{fu}^{LoS}\left(t\right)+P^{NLoS}\left(t\right)L_{fu}^{NLoS}\left(t\right).$$

The received power $p_{fu}$ between GBU *u* and FBS *f* for a transmit power $p_{t}$ at time slot *t* can be calculated as:

(6) $$p_{fu}\left(t\right)=p_{t}-L_{fu}\left(t\right).$$

Equation (6) applies to both uplink and downlink transmission.

### 3.3. Association Model

For our network scenario, we consider separate uplink and downlink transmissions. We introduce a binary variable β to indicate whether a given FBS is serving a GBU at a given time slot *t* for either uplink or downlink transmission.

The binary variable $\beta_{fu}^{↑}$ at timeslot *t* is defined as:

(7) $$\beta_{fu}^{↑}\left(t\right)=\left\{\begin{array}{cc} 1, & \mathrm{if}\;\mathrm{GBU}\;u\;\mathrm{is}\;\mathrm{associated}\;\mathrm{with}\;\mathrm{FBS}\;f\;\mathrm{for}\;\mathrm{UL},\\ 0, & \mathrm{otherwise}. \end{array}\right.$$

Similarly, the binary variable $\beta_{fu}^{↓}$ is defined as:

(8) $$\beta_{fu}^{↓}\left(t\right)=\left\{\begin{array}{cc} 1, & \mathrm{if}\;\mathrm{GBU}\;u\;\mathrm{is}\;\mathrm{associated}\;\mathrm{with}\;\mathrm{FBS}\;f\;\mathrm{for}\;\mathrm{DL},\\ 0, & \mathrm{otherwise}. \end{array}\right.$$

A GBU can be associated with only one FBS for uplink transmission and one FBS for downlink transmission in a given time slot *t*. Thus, the following constraints ensure this condition:

(9) $$\sum_{u=1}^{U}\beta_{fu}^{↑}\left(t\right)=1,$$

(10) $$\sum_{u=1}^{U}\beta_{fu}^{↓}\left(t\right)=1.$$

### 3.4. Data Rate

The performance of the network is measured by the achievable data rate of transmission links between FBSs and GBUs. The data rates for uplink and downlink transmissions differ due to varying levels of interference at FBSs and GBUs.

#### 3.4.1. Uplink Transmission

The theoretical uplink data rate $r_{fu}^{↑}$ between GBU *u* and FBS *f* at time slot *t* can be calculated using Shannon–Hartley’s equation as follows:

(11) $$r_{fu}^{↑}\left(t\right)=B\log_{2}\left(1+\gamma_{fu}^{↑}\left(t\right)\right),$$

where $\gamma_{fu}^{↑}$ is the signal-to-interference-plus-noise ratio (SINR) of the uplink channel between GBU *u* and FBS *f* and is given by:

(12) $$\gamma_{fu}^{↑}\left(t\right)=\frac{p_{fu}\left(t\right)}{I_{f}\left(t\right)+\sigma^{2}}.$$

Here, $\sigma^{2}$ represents the cumulative effect of external interference and additive white Gaussian noise (assumed to be the same for all devices), and $I_{f}$ is the interference received by FBS *f* at time slot *t*. The interference $I_{f}\left(t\right)$ at FBS *f* while communicating with GBU *u* at time slot *t* is the sum of the received power from all other nodes (both FBSs and GBUs) using the same channel for communication and the self interference:

(13) $$I_{f}\left(t\right)=\sum_{i=1,i\neq f}^{F}\phi_{m}\left(t\right)I_{i}\left(t\right)+\sum_{j=1,j\neq u}^{U}\phi_{m}\left(t\right)I_{j}\left(t\right)+I_{f}^{self},$$

where $\phi_{m}\left(t\right)$ is a binary variable indicating whether the device is utilizing the same channel as FBS *f* at time slot *t*.

#### 3.4.2. Downlink Transmission

Similar to the uplink data rate, the downlink data rate can be calculated as:

(14) $$r_{uf}^{↓}\left(t\right)=B\log_{2}\left(1+\gamma_{uf}^{↓}\left(t\right)\right),$$

where $\gamma_{uf}^{↓}$ is the SINR of the downlink channel between GBU *u* and FBS *f*, and is given by:

(15) $$\gamma_{uf}^{↓}\left(t\right)=\frac{p_{fu}\left(t\right)}{I_{u}\left(t\right)+\sigma^{2}}.$$

Here, $I_{u}\left(t\right)$ represents the interference level at GBU *u* on channel *m*, which is the sum of the signal power received at GBU *u* from all nodes transmitting on the same channel *m* and the self interference:

(16) $$I_{u}\left(t\right)=\sum_{k=1,k\neq f}^{F}\phi_{m}\left(t\right)I_{k}\left(t\right)+\sum_{l=1,l\neq u}^{U}\phi_{m}\left(t\right)I_{l}\left(t\right)+I_{u}^{self}.$$

The maximum data rate that an FBS can handle in time slot *t* is limited by the capacity of the backhaul communication link between the FBS and GBS and can be calculated as:

(17) $$r_{f}^{G}=B\log_{2}\left(1+\gamma_{Gf}\right),$$

where $\gamma_{Gf}$ is the SINR of the backhaul link between FBS *f* and GBS.

### 3.5. Problem Formulation

Our main objective is to maximize the network’s data rate over a given time period *T* by optimizing UAV trajectories and GBU associations. We formulate the problem as follows:

(18)P1maxβ,l,p1T∑t=1T∑f=1F∑u=1Urfu↑(t)+∑t=1T∑f=1F∑u=1Urfu↓(t),subjectto(18a)∑u=1Uβfu↑(t)=1,∑u=1Uβfu↓(t)=1,∀f∈F,u∈U,(18b)∑u=1Uβfu↑rfu↑(t)+∑u=1Uβfu↓rfu↓(t)≤rfG,∀f∈F,(18c)||lf(t)−lf′(t)||≥dmin,∀f≠f′∈F,(18d)1T∑f∈Fpf(t)≤pfavg,∀f∈F,(18e)lf(1)=lf(T),∀f∈F.

The objective function defined in Equation (18) aims to optimize the average combined uplink and downlink data rates of the network. Constraint (18a) ensures that each GBU is associated with exactly one FBS for uplink transmission and one FBS for downlink transmission. Constraint (18b) limits the total data rate of associated GBUs to the maximum data rate of the link between the FBS and the GBS. Constraint (18c) enforces a minimum distance between UAVs to avoid collisions. Constraint (18d) limits the total transmit power of each FBS to its average power in each time slot. Constraint (18e) ensures that each UAV returns to its starting point at the end of the time period *T*.

The complexity of the formulated problem, characterized by high-dimensional state and action spaces, dynamic constraints, and multiple interacting variables, renders heuristic methods inadequate. Specifically, the state space complexity is O((F×U)×T), the action space complexity is $O({\left(F\times U\right)}^{T})$, and constraint checking adds further complexity of O(F×U). These factors collectively challenge heuristic methods due to their limited capacity to explore and evaluate the vast solution space effectively. Heuristics also struggle with their reliance on approximate strategies, which do not guarantee optimal solutions and are less scalable as network size increases. In contrast, Distributed Deep Reinforcement Learning (DRL) is well-suited to address these challenges, offering robust performance by efficiently managing large-scale, complex problems, adapting to real-time changes, and optimizing policies continuously. This makes DRL a more effective and scalable approach for solving the formulated problem compared to traditional heuristic methods.

## 4. Proposed Solution

In this article, our main focus is to maximize the overall data rate of the network by optimizing the FBS trajectory and GBUs association for uplink and downlink transmissions. We propose the Multi-Agent Deep Deterministic Policy Gradient (MADDPG) based solution for our problem. In the proposed DRL-based approach each FBS (UAV) acts as an agent which interacts with the environment to gather the local information. We first transform the problem into a partially observable Markov Decision Process (POMDP) and then use the centralized training and distributed inference by the trained model. The UAVs can only observe a part of the environment thus leading to uncertainty due to limited information. Therefore, during the centralized training phase, the UAVs jointly learn the policies. Specifically, we define the state space S for each UAV, which includes information such as the positions of the UAVs and ground users, current channel states, and trajectory details. The action space A encompasses decisions related to trajectory adjustments, power control, and user association. We formulate the reward function R to capture the objectives of maximizing data rates and minimizing interference. Transition dynamics P describe how states evolve based on actions, while the observation space O reflects the partial observability of each UAV. Using MADDPG, we train a centralized critic to evaluate joint actions and decentralized actors to make decisions based on local observations, thus facilitating efficient multi-agent coordination and distributed execution.

### 4.1. Problem Transformation into POMDP

In our problem, UAVs functioning as flying base stations are considered agents that make decisions based on their local observations. In the formulated problem in Equation (18), the UAVs behavior is influenced by the network environment, given their limited sensing and communication range, these agents can only partially observe the environment, leading to a scenario where they possess only local information rather than full global knowledge. This situation can be effectively modeled using a Partially Observable Markov Decision Process (POMDP). The POMDP is represented by the tuple $\left(S,A,R,O,P^{T},P^{O},\delta \right)$, where S denotes the set of possible states, A represents the set of actions available to the agents, R is the reward function, O is the set of observations, $P^{T}$ is the state transition function, $P^{O}$ defines the observation function, and δ is the discount factor. This model helps in capturing the uncertainty and partial observability inherent in the UAVs’ operational environment.

- **State Space S**: The state $s_{t}$ at a given time slot *t* encapsulates the entire system configuration, including the positions, velocities, user associations, and transmission power levels of all UAVs. Formally, the state $s_{t}$ at time *t* can be written as:
  (19) $$s_{t}=\left\{l_{f}\left(t\right),\nu_{f}\left(t\right),r_{f}^{G}\left(t\right),r_{fu}^{↑}\left(t\right),r_{fu}^{↓}\left(t\right)\right\},f\in F,$$
  where $l_{f}\left(t\right)$ is the position of UAV *f*, $\nu_{f}\left(t\right)$ is its velocity, and $r_{f}^{G}\left(t\right),r_{fu}^{↑}\left(t\right),r_{fu}^{↓}\left(t\right)$ represent the capacity and uplink/downlink data rates of the associated GBUs. The complete state space S contains all such possible states.
- **Observation Space O** Due to partial observability, each UAV can only perceive local information, represented as an observation $o_{f}\left(t\right)$, which includes its position, velocity, and channel quality indicators for the GBUs in its vicinity. This observation is a subset of the global state, such that:
  (20) $$o_{t}^{f}=\left\{l_{f}\left(t\right),\nu_{f}\left(t\right),r_{f}^{G}\left(t\right),\gamma_{fu}^{↑}\left(t\right),\gamma_{fu}^{↓}\left(t\right)\right\}.$$
  where $\gamma_{fu}^{↑}\left(t\right)$ and $\gamma_{fu}^{↓}\left(t\right)$ denote the signal-to-noise ratios for uplink and downlink. The observation space O for each UAV reflects this limited view.
- **Action Space A**: The actions of the UAVs involve adjusting their velocities, user associations, and transmission power. The action $a_{f}\left(t\right)$ of UAV *f* at time *t* can be described as:
  (21) $$a_{t}=\left\{\beta_{f}^{↑}\left(t\right),\beta_{f}^{↓}\left(t\right),\Phi_{f}\left(t\right),\nu_{f}\left(t\right),p_{f}\left(t\right)\right\},$$
  where $\beta_{f}^{↑}\left(t\right)$ and $\beta_{f}^{↓}\left(t\right)$ denote uplink and downlink associations, $\Phi_{f}\left(t\right)$ represents the flying direction, $\nu_{f}\left(t\right)$ is the velocity, and $p_{f}\left(t\right)$ is the transmission power. The joint action space for all UAVs forms the set A.
- **Reward R(s,a)**: The reward function is designed to reflect the network performance by considering data rate maximization and penalty functions to ensure that constraints are met. The reward at time *t* is as follows:
  (22) $$R_{t}=r\left(t\right)-\varphi_{1}\kappa^{capacity}-\varphi_{2}\kappa^{bound}-\varphi_{3}\kappa^{power},$$
  where r(t) represents the total data rate, and $\kappa^{\mathrm{capacity}},\kappa^{\mathrm{bound}},\kappa^{\mathrm{power}}$ are penalty terms for violating capacity, boundary, and power constraints, respectively. The weights $\varphi_{1},\varphi_{2},\varphi_{3}$ control the significance of these penalties.
- **Observation Function $P^{O}\left(o|s,a\right)$**: Since each FBS only has partial observability, the observation function $P^{O}\left(o_{f}^{t}|s_{t},a_{s}^{t}\right)$ represents the probability of FBS *f* observing $o_{f}^{t}$ given the current state $s_{t}$ and action $a_{f}^{t}$.
- **State Transition Function $P^{T}\left(s,a,s^{\prime }\right)$**: The state transition function $P^{T}\left(s,a,s^{\prime }\right)=P\left(s_{t+1}|s_{t},a_{t}\right)$ defines the probability of transitioning from state $s_{t}$ to state $s_{t+1}$ given the action $a_{t}$. This captures the dynamics of UAV movement, user association changes, and power level adjustments: Consider a sequence of states and actions $S_{T}=\left\{s_{1},a_{1},s_{2},a_{2},\ldots ,s_{T},a_{T}\right\}$. The probability of this sequence is given by the following:
  (23) $$P\left(S_{T}\right)=P\left(s_{1}\right)\prod_{t=1}^{T}\pi \left(a_{t}|s_{t}\right)\cdot \mathrm{Pr}\left(s_{t+1}|s_{t},a_{t}\right),$$
  where $\pi (a_{t}|s_{t})$ represents the policy that dictates the probability of taking action $a_{t}$ given state $s_{t}$, and $\mathrm{Pr}(s_{t+1}|s_{t},a_{t})$ is the transition probability to the next state $s_{t+1}$.
  The transition probability $\mathrm{Pr}(s_{t+1}|s_{t},a_{t})$ can be computed by integrating over all possible future states $s_{t+1}$ as follows:
  (24) $$\mathrm{Pr}\left(s_{t+1}|s_{t},a_{t}\right)=\int_{S^{\prime }}\lambda \left(s_{t},a_{t},s_{t+1}\right),ds_{t+1},$$
  where $\lambda (s_{t},a_{t},s_{t+1})$ denotes the probability density function representing the likelihood of transitioning from state $s_{t}$ to $s_{t+1}$ when action $a_{t}$ is taken.

### 4.2. MADDPG Based Algorithm

To solve the formulated POMDP problem, we employ the Multi-Agent Deep Deterministic Policy Gradient (MADDPG) algorithm. Given the decentralized nature of the UAVs and the partial observability of the environment, MADDPG is well-suited for scenarios where agents (UAVs in our case) must make decisions based on local observations while learning in a centralized manner. In this study, we emphasize MADDPG’s efficacy in optimizing user associations and UAV trajectories within a POMDP framework, allowing for efficient real-time decision-making. This approach is particularly well-suited for dynamic scenarios, such as disaster response, where adaptability is crucial.

MADDPG builds on the Deep Deterministic Policy Gradient (DDPG) algorithm by adapting it to multi-agent settings. Each UAV uses an actor network to map its local observations to actions, while the training phase remains centralized, supported by a global critic network that evaluates actions using joint state and action information from all agents. This centralized training helps mitigate issues like non-stationarity and partial observability, which are key challenges in the multi-agent environment modeled by the POMDP.

**Figure 3:**
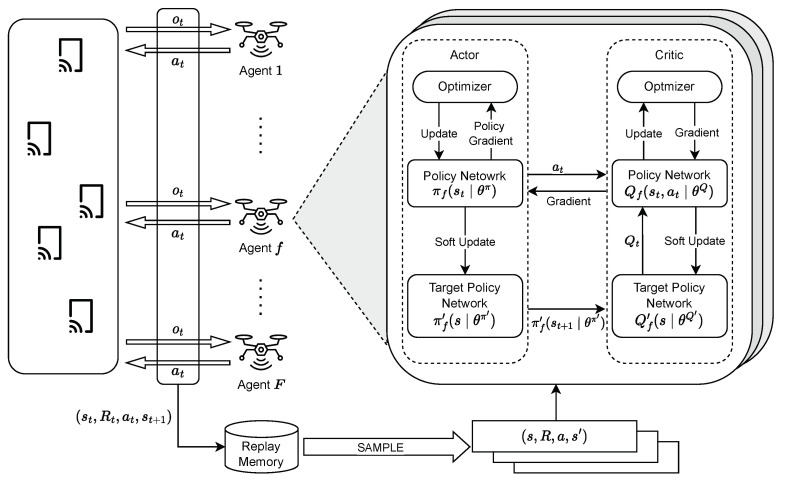
MADRL framework for Multi-UAV network.

The key advantage of MADDPG lies in its ability to allow decentralized execution. After training, each UAV relies solely on its local observations to make decisions, while the centralized critic is only used during the learning process. This decentralized execution is essential in large-scale environments where global information is inaccessible to individual UAVs during operation. Figure 3 depicts the MADDPG-based multi-agent network environment.

### 4.3. Centralized Training

During the training phase, a central controller collects the joint observations, actions, and rewards from all UAVs. This enables centralized training, where each agent’s policy is learned based on the global state and action information of the entire network. For each agent *f*, the critic evaluates the state-action value function $Q_{f}\left(s_{t},a_{t}\right)$, representing the expected cumulative reward for taking action $a_{t}$ in state $s_{t}$:

(25) $$Q_{f}\left(s_{t},a_{t}\right)=E_{s^{\prime }∼P\left(s^{\prime }|s,a\right)}\left[r_{f}\left(s,a\right)+\gamma \max_{a’}Q_{f}\left(s^{\prime },a^{\prime }\right)\right],$$

where the joint action $a_{t}={a_{f}\left(t\right)}_{f=1}^{F}$ includes the actions of all agents and the next state $s^{\prime }$ is derived from the state transition function $P(s^{\prime }|s,a)$. The critic is updated by minimizing the temporal difference (TD) error through the Bellman equation:

(26) $$L\left(\theta_{f}^{Q}\right)=E_{\left(s,a,r,s^{\prime }\right)}\left[{\left(r_{f}+\Gamma Q_{f}\left(s^{\prime },a^{\prime };\theta_{f}^{Q}\right)-Q_{f}\left(s,a;\theta_{f}^{Q}\right)\right)}^{2}\right],$$

where $\theta_{f}^{Q}$ represents the parameters of the critic network and $\theta_{f}^{Q^{\prime }}$ represents the parameters of the target critic network and Γ is the discount factor.

### 4.4. Target Network Update

To stabilize training, target networks are introduced for both the actor and critic networks. These target networks are updated slowly to ensure gradual changes during training. The target network weights are updated using the following soft update rule after each time step:

(27) $$\theta_{f}^{Q^{\prime }}\leftarrow \tau \theta_{f}^{Q}+\left(1-\tau \right)\theta_{f}^{Q^{\prime }},$$

(28) $$\theta_{f}^{\pi^{\prime }}\leftarrow \tau \theta_{f}^{\pi }+\left(1-\tau \right)\theta_{f}^{\pi^{\prime }},$$

where τ is a small factor (e.g., 0.001), controlling the rate of change. This ensures that the target networks evolve smoothly, preventing drastic updates that could destabilize learning.

### 4.5. Decentralized Execution

Once training is complete, each UAV can execute its learned policy in a decentralized manner, relying solely on its local observations. The actor network for agent *f* is parameterized by $\theta_{f}^{\pi }$ and outputs actions based on the current observation $o_{f}\left(t\right)$:

(29) $$a_{f}\left(t\right)=\pi_{f}\left(o_{f}\left(t\right);\theta_{f}^{\pi }+N_{t}\right),$$

where $N_{t}$ represents the exploration noise, typically modeled using the Ornstein-Uhlenbeck process to generate temporally correlated exploration for smoother action sequences. The noise ensures that the agent does not converge prematurely to a suboptimal policy by encouraging the exploration of diverse actions. The policy is updated by maximizing the expected return:

(30) $$J\left(\pi_{f}\right)=E_{s_{t},a_{t}∼\pi_{f}}\left[Q_{f}\left(s_{t},a_{t}\right)\right].$$

The gradients of the actor’s policy are computed using the deterministic policy gradient (DPG) algorithm:

(31) $$\nabla_{\theta_{f}^{\pi }}J\left(\pi_{f}\right)=E\left[\nabla_{\theta_{f}^{\pi }}\pi_{f}\left(o_{f}\right)\nabla_{a_{f}}Q_{f}(s_{t},a_{t})\right].$$

To improve learning efficiency and reduce the correlation between samples, we use experience replay. Each agent stores its experiences $\left(s_{t},a_{t},r_{t},s_{t+1}\right)$ in a replay buffer. Random batches of experiences are sampled from this buffer during training to update both the actor and critic networks. This helps the agents to learn from past experiences and break the correlation between sequential updates. The detailed steps of the MADDPG algorithm for UAV trajectory design and user association are provided in Algorithm 1. The algorithm starts by initializing actor and critic networks with random weights for each UAV, followed by the initialization of target networks and a replay buffer. In each episode, the environment is reset, and the initial state is retrieved (line 2). At each time step, UAVs observe their local states and select actions with added exploration noise (lines 4–6). The joint actions are executed, and the next state and rewards are obtained (line 8). Transitions are stored in the replay buffer (line 9). The critic networks are updated by minimizing the loss function using a randomly sampled minibatch (lines 10, 11), while the actor networks are updated via the policy gradient (lines 13–14). Lastly, the target networks are softly updated (lines 17–18).
**Algorithm 1** MADDPG for UAV Trajectory Design and User Association**Require:** 
Initialize actor network $\pi_{f}\left(o_{f};\theta_{f}^{\pi }\right)$ and critic network $Q_{f}\left(s,a;\theta_{f}^{Q}\right)$ with random weights for each UAV *f***Require:** 
Initialize target networks $\theta_{f}^{\pi^{\prime }}\leftarrow \theta_{f}^{\pi }$ and $\theta_{f}^{Q^{\prime }}\leftarrow \theta_{f}^{Q}$**Require:** 
Initialize replay buffer D  1:**for** each episode **do**  2:      Reset the environment and obtain initial state $s_{0}$  3:      **for** each time step *t* **do**  4:            **for** each UAV *f* **do**  5:                  Observe local observation $o_{f}\left(t\right)$  6:                  Select action $a_{f}\left(t\right)=\pi_{f}\left(o_{f}\left(t\right);\theta_{f}^{\pi }\right)+N_{t}$ (with exploration noise $N_{t}$)  7:            **end for**  8:            Execute joint action $a_{t}={\left\{a_{f}\left(t\right)\right\}}_{f=1}^{F}$ and observe reward $r_{t}$ and next state $s_{t+1}$  9:            Store transition $\left(s_{t},a_{t},r_{t},s_{t+1}\right)$ in replay buffer D10:            **for** each UAV *f* **do**11:                  Sample a random minibatch of *N* transitions $\left(s,a,r,s^{\prime }\right)$ from D12:                  Compute target value: $y=r_{f}+\gamma Q_{f}\left(s^{\prime },a^{\prime };\theta_{f}^{Q^{\prime }}\right),$ where $a^{\prime }={\left\{\pi_{f}\left(o_{f}^{\prime };\theta_{f}^{\pi^{\prime }}\right)\right\}}_{f=1}^{F}$13:                  Update critic by minimizing the loss: $L\left(\theta_{f}^{Q}\right)=\frac{1}{N}\sum {\left(y-Q_{f}\left(s,a;\theta_{f}^{Q}\right)\right)}^{2}$14:                  Update actor using the sampled policy gradient:                  $\nabla_{\theta_{f}^{\pi }}J\left(\pi_{f}\right)=\frac{1}{N}\sum \nabla_{a_{f}}Q_{f}\left(s,a;\theta_{f}^{Q}\right)\nabla_{\theta_{f}^{\pi }}\pi_{f}\left(o_{f};\theta_{f}^{\pi }\right)$15:            **end for**16:            Update target networks:17:            $\theta_{f}^{Q^{\prime }}\leftarrow \tau \theta_{f}^{Q}+\left(1-\tau \right)\theta_{f}^{Q^{\prime }}$18:            $\theta_{f}^{\pi^{\prime }}\leftarrow \tau \theta_{f}^{\pi }+\left(1-\tau \right)\theta_{f}^{\pi^{\prime }}$19:      **end for**20:**end for**

## 5. Algorithm Validation and Analysis

### 5.1. Experimental Design and Parameters

The performance of the MADDPG algorithm for UAV trajectory design and user association was evaluated through a series of simulations. The system comprised five UAVs functioning as FBSs and 15 GBUs. These entities were distributed in a 1000 × 1000 m square area. Communication was modeled using a full-duplex mode where uplink and downlink channels were independently managed. The simulations utilized a bandwidth of 20 MHz and UAVs operated with a transmit power of 30 dBm. The external noise power was set at −60 dB, and the channel gain was modeled using a path loss exponent of 4 and a reference path loss of 30 dB. The MADDPG algorithm employed deep neural networks with two hidden layers, each containing 64 neurons, for both actor and critic networks. Exploration was facilitated by an Ornstein-Uhlenbeck process with mean reversion level = 0.15, volatility parameter = 0.2 and mean reversion rate = 0.1.

The training involved a replay buffer of 1,000,000 transitions and a batch size of 256. The discount factor was set to 0.99, and the soft target update rate was 0.01. The algorithm was trained over 5000 episodes, with each episode lasting 1000 time steps. Learning rates were 0.001 for the actor network and 0.001 for the critic network. The simulations were carried out using Python with TensorFlow for the implementation of the MADDPG algorithm. The experiments were executed on a computing system equipped with an Intel Core i7 processor, 16 GB of RAM, and an NVIDIA GTX 1080 GPU, with data storage managed by a 1 TB SSD. The rest of the system parameters are given in Table 1.

### 5.2. Simulation Results and Interpretation

In the first experiment, we assessed the capability of our scheme to manage the association of GBUs with FBSs (UAVs) for both uplink and downlink channels independently. The results, detailed in Table 2, illustrate the association of 15 GBUs with five UAVs across four time slots. The experiment demonstrates that our scheme efficiently handles simultaneous uplink and downlink associations, allowing GBUs to connect to different UAVs (FBSs) for each communication direction. This dual-mode management ensures effective communication and resource utilization in the full-duplex system.

**Figure 4:**
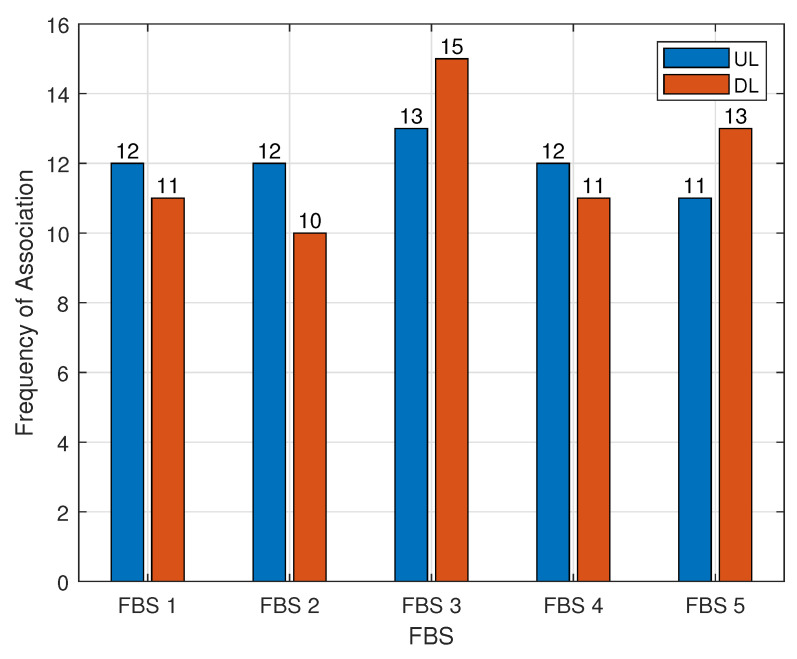
Frequency of association of each FBS during four timeslots.

Figure 4 depicts the number of GBUs associated with each FBS (UAV) for both uplink and downlink channels over the four time slots. The data shows a relatively consistent number of associations across different FBSs, with slight variations between uplink and downlink connections. Specifically, FBS 3 exhibits the highest number of GBU associations in both uplink (13) and downlink (15) modes, suggesting a more central role or higher demand in the network. Conversely, FBS 5 shows the lowest number of uplink associations (11) but a higher number of downlink associations (13), indicating a possible shift in resource allocation or GBU preference. Overall, the uniform distribution of GBUs across FBSs illustrates the effectiveness of our scheme in maintaining balanced and flexible GBU-FBS associations in the full-duplex system. These results demonstrate the effectiveness of the proposed decoupling strategy, which ensures flexible resource allocation between UAVs. The balance of associations across the UAVs indicates that the network can dynamically adapt to varying data traffic demands without overwhelming specific FBSs.

**Figure 5:**
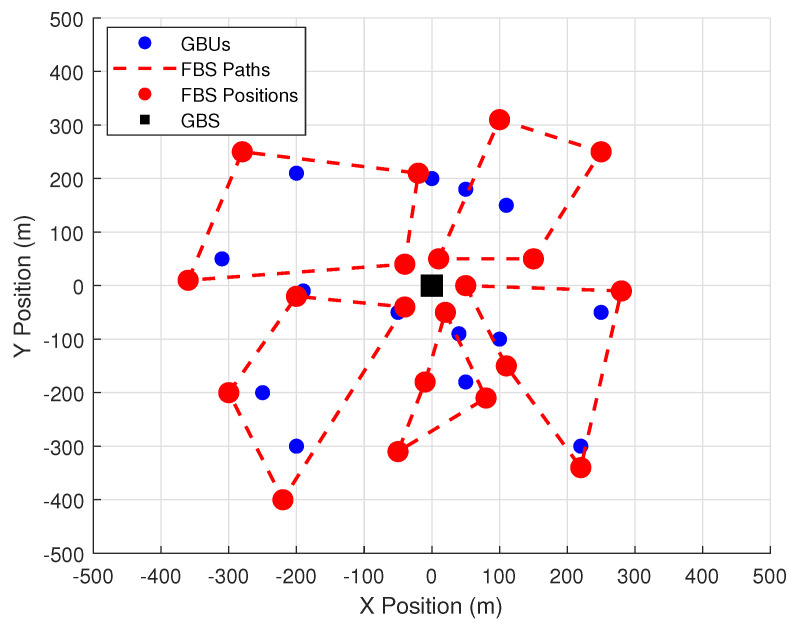
UAV flight trajectories during time *T*.

Figure 5 illustrates the flight paths of the FBSs. The initial positions of the FBSs are set near the GBS and the altitude for this experiment is fixed at 180 m. As the figure shows, the FBSs carefully navigate their paths to maximize the coverage of the GBUs, adjusting their trajectories dynamically to avoid interference and collisions. The FBSs maintain safe separation distances, ensuring optimal performance without overlapping coverage areas. Furthermore, the FBSs follow closed-loop trajectories, meaning they successfully return to their initial positions, demonstrating the efficiency of the path-planning algorithm in ensuring full coverage and trajectory closure while minimizing energy consumption. The dynamic adjustments in trajectory highlight the flexibility of the UAVs in maintaining optimal coverage in changing network environments. These adjustments lead to improved resource allocation and interference mitigation, directly affecting the network’s ability to serve more GBUs efficiently.

**Figure 6:**
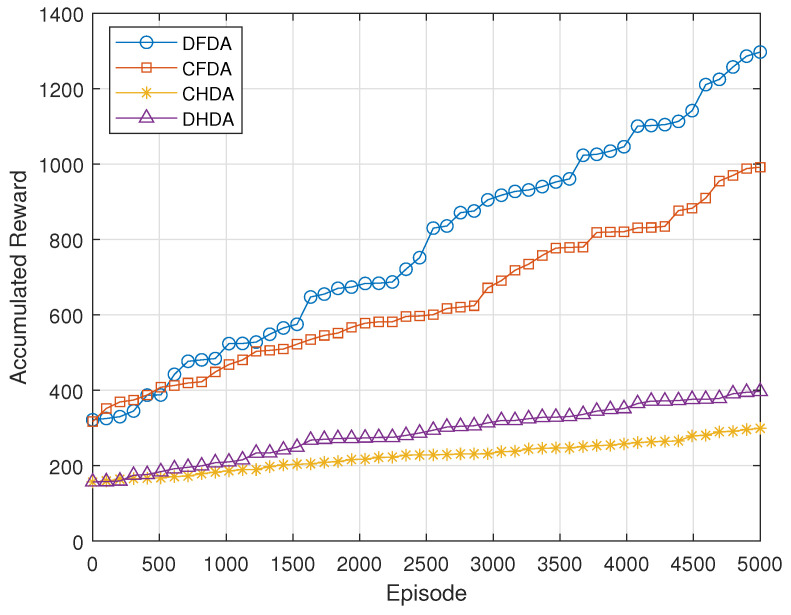
Accumulated Reward based on different association methods.

Figure 6 illustrates the accumulated reward for various association methods namely:

- **CHDA:** Coupled Half-Duplex Association, where GBUs are associated with the same UAV for both uplink and downlink transmissions in a Half-Duplex mode.
- **DHDA:** Decoupled Half-Duplex Association, where GBUs are connected to different UAVs for uplink and downlink transmissions, still operating in a Half-Duplex mode.
- **CFDA:** Coupled Full-Duplex Association, in which GBUs associate with the same UAV for both uplink and downlink transmissions in a Full-Duplex mode.
- **DFDA (proposed):** Decoupled Full-Duplex Association, where GBUs are connected to separate UAVs for uplink and downlink transmissions in a Full-Duplex mode.

The proposed DFDA mode achieves the highest reward, surpassing the coupled full-duplex approach. This is because, in DFDA, GBUs can connect to different UAVs for uplink and downlink transmissions, allowing for better optimization of each link individually. By decoupling the associations, DFDA minimizes interference and allows for more flexible resource allocation between UAVs, which leads to improved spectral efficiency and reduced transmission delays. In contrast, coupled full-duplex and half-duplex modes are more constrained, either by forcing the GBU to use the same UAV for both directions or by operating in a less efficient half-duplex mode. These results clearly demonstrate the superiority of DFDA in efficiently managing resources and reducing interference, leading to higher overall network performance. The DFDA method’s superiority over the coupled and half-duplex methods highlights the significance of decoupling associations in a full-duplex setting. This approach enhances spectral efficiency, reduces transmission delays, and optimizes overall network performance compared to existing schemes. The performance improvement is particularly notable under high traffic and interference conditions, showing that DFDA can better handle real-world network demands.

**Figure 7:**
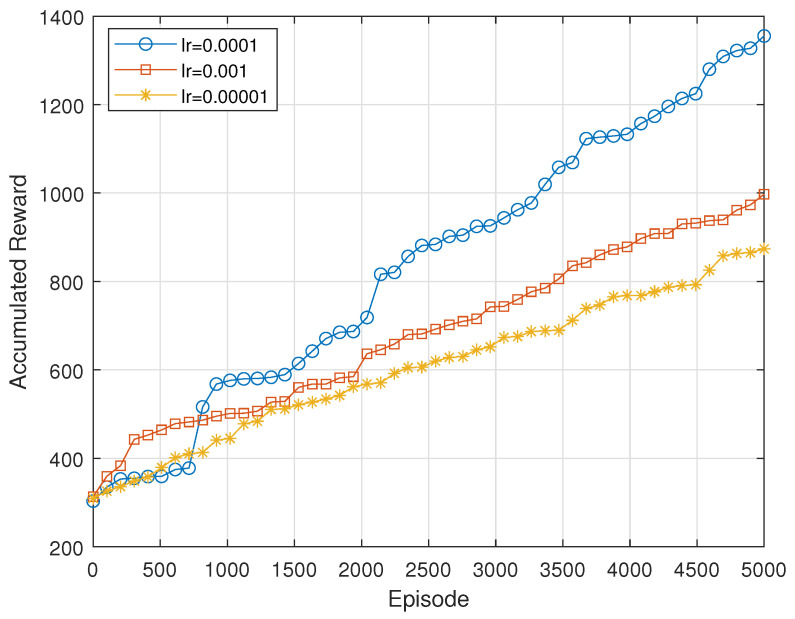
Accumulated reward with different learning rates.

Figure 7 shows the accumulated reward over episodes for different learning rates (0.001, 0.0001, and 0.00001). The learning rate of 0.001 starts slowly but achieves the highest reward, indicating effective long-term learning and convergence. The rate of 0.0001 shows quick initial gains but falls short of 0.001 in the long run, suggesting that it may lead to instability or suboptimal learning. The rate of 0.00001 results in the lowest reward, reflecting its overly conservative nature that hinders effective learning and convergence.

**Figure 8:**
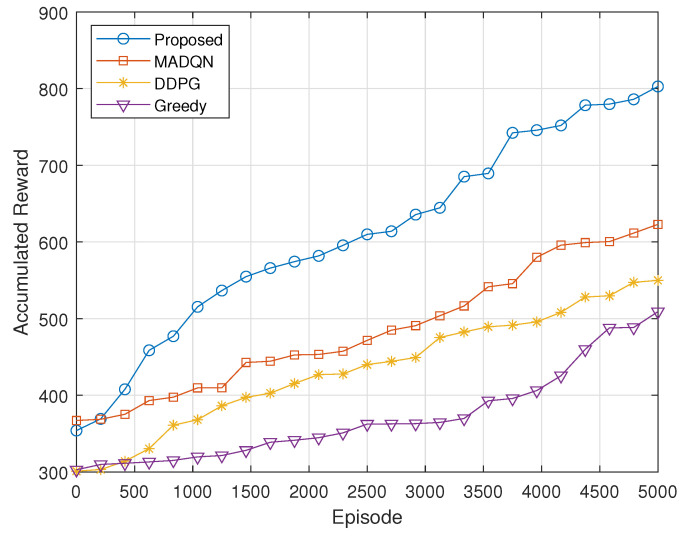
Comparison of proposed algorithm with different algorithms.

Figure 8 illustrates the accumulated reward obtained using the proposed algorithm in comparison to MADQN, DDPG, and Greedy. The proposed algorithm achieves the highest accumulated reward, demonstrating its superior capability in optimizing the communication system’s performance. In contrast, MADQN, while effective, yields a lower accumulated reward than the proposed algorithm, suggesting its performance is not as optimal. DDPG and Greedy show progressively lower accumulated rewards, indicating that these methods are less effective in this context. This result highlights the proposed algorithm’s enhanced effectiveness in maximizing accumulated rewards compared to the other algorithms evaluated. The superior performance of the proposed algorithm demonstrates its advantage in addressing complex multi-agent environments like UAV-assisted networks. In comparison, existing algorithms such as MADQN and DDPG struggle to optimize the system effectively, particularly in dynamic and large-scale settings where decentralized execution is key.

**Figure 9:**
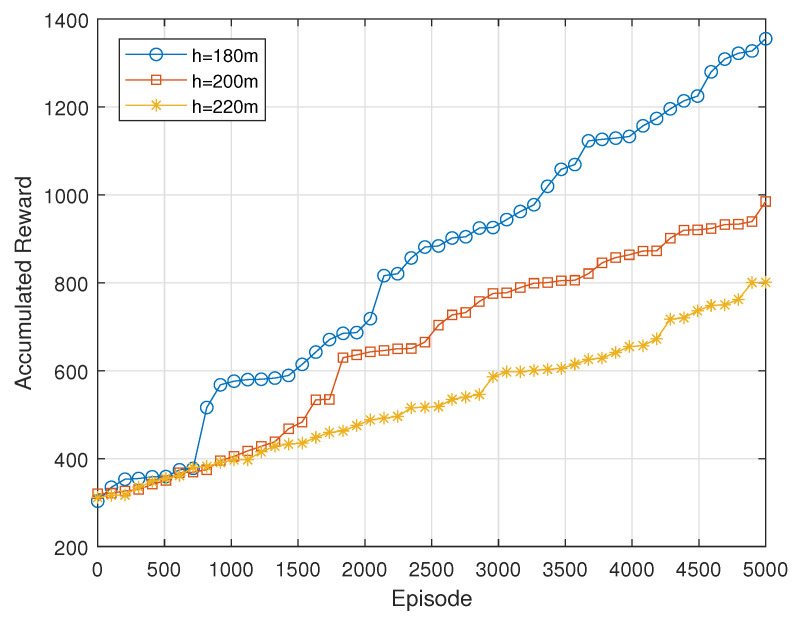
Accumulated reward with different heights of UAVs.

Figure 9 illustrates the accumulated reward achieved with varying UAV flight heights: 180 m, 200 m, and 220 m. The flight height of 180 m results in the highest accumulated reward, indicating that this height provides an optimal balance between coverage and signal quality. The height of 200 m yields moderate performance, suggesting that it may offer less effective coverage or signal strength compared to 200 m. Conversely, the flight height of 220 m results in the lowest accumulated reward, likely due to diminished signal quality. These results highlight the importance of selecting an optimal flight height to maximize performance in UAV-based communication systems.

## 6. Conclusions

In conclusion, this paper presents a novel approach for improving the sum rate in full-duplex UAV-assisted communication networks by decoupling uplink and downlink user associations. By formulating the problem as a POMDP and solving it through the MADDPG algorithm, we have demonstrated the effectiveness of using multi-agent deep reinforcement learning (MADRL) in achieving optimal user association and UAV trajectory control. Our results clearly show that the proposed DFDA method outperforms other association schemes, achieving the highest accumulated reward by reducing interference and allowing for more efficient resource allocation.

Additionally, our solution optimizes UAV trajectories and flight heights to ensure efficient coverage and minimal energy consumption. This framework, which combines centralized training with distributed execution, proves superior to existing methods in terms of flexibility, scalability, and adaptability, especially in real-world critical applications like disaster response and search and rescue missions. The practical value of our approach lies in its ability to rapidly deploy UAV networks while maintaining high operational efficiency. Future work can explore extending this approach to more complex network scenarios and incorporating additional real-world constraints.

## Figures and Tables

**Table 1:** Proposed Parameter Settings.

Parameters	Values
Number of GBS (G)	01
Number of FBS (F)	05
Number of GBUs (U)	15
Height of FBS (*h*)	180 m
Maximum flight velocity of FBS ($\nu_{f}^{max}$)	20 m/s
Maximum Power of FBS ($p_{f}^{max}$)	30 dBm
Maximum Power of GBU ($p_{u}^{max}$)	30 dBm
External noise level $\sigma^{2}$	−60 dBm
Self-interference cancellation χ	100 dB
Attenuation factors ${ℵ}^{LoS},{ℵ}^{NLoS}$	1.44544, 199.526
Carrier frequency (μ)	2 GHz
Weights ($\kappa_{1},\kappa_{2},\kappa_{3}$)	50, 500, 50
Learning rate	0.0001
Discount factor (Γ)	0.99

**Table 2:** GBU association with FBS where No. of FBSs is Four and No. of GBUs is 15.

	GBU	U1	U2	U3	U4	U5	U6	U7	U8	U9	U10	U11	U12	U13	U14	U15
T1	UL	2	1	4	5	3	2	3	3	1	3	5	1	3	4	2
	DL	5	3	3	1	5	2	4	5	4	3	3	1	3	4	5
T2	UL	2	4	1	5	3	2	5	4	3	1	2	4	1	5	3
	DL	3	1	4	2	5	4	3	2	5	1	4	3	5	2	1
T3	UL	3	1	5	4	2	3	1	4	5	2	3	5	1	4	2
	DL	2	4	3	1	5	4	2	5	3	1	4	2	5	3	1
T4	UL	1	5	3	4	2	5	1	3	4	2	5	3	1	4	2
	DL	4	2	5	1	3	4	2	5	1	3	4	2	5	1	3

## Data Availability

Data are contained within the article.
